# Les manifestations cutanées au cours du COVID-19: état des lieux

**DOI:** 10.11604/pamj.supp.2020.35.132.24842

**Published:** 2020-08-04

**Authors:** Madiha El Jazouly, Fatim zahra Chahboun, Soumiya Chiheb

**Affiliations:** 1 Service de dermatologie, Hôpital Cheikh khalifa Ibn Zaid, Université Mohamed VI des sciences de la santé de Casablanca, Maroc

**Keywords:** Manifestations cutanées, COVID-19, revue

## To the editors of the Pan African Medical Journal

L´infection COVID-19 ou *Coronavirus disease* 19 est une maladie infectieuse émergente et particulière, à la fois sur le plan physiopathologique, clinique et thérapeutique. Depuis son apparition en Chine, une répartition mondiale a été déclarée avec une épidémiologie en constante évolution. La transmission interhumaine de ce nouveau coronavirus ou SARS-COV-2 se fait surtout via des gouttelettes respiratoires. Ainsi le virus a été mis en évidence dans différents sites du corps avec une prédominance au niveau des alvéoles pulmonaires dans 93% des cas. Ceci est en rapport avec la présence des récepteurs au virus de type ACE 2 au niveau des voies aériennes supérieures et inférieures, au niveau digestif, cardiaque, cérébral et vasculaire expliquant le tableau clinique très varié de cette infection allant d´un syndrome grippal paucisymptomatique à une pneumopathie hypoxemiante avec défaillance multi viscérale [1]. Le bilan biologique peut également être très perturbé se rapprochant d´un syndrome d'activation macrophagique. En effet, caractérisée par un choc cytokinique, la COVID-19 est loin d'être une simple pneumonie virale mais une pneumonie ayant un tropisme pulmonaire et endothélial entrainant une vasculite multifocale. Par ailleurs la réponse de l´hôte au virus dépend du terrain, ainsi le retentissement de cet état inflammatoire est régenté chez le sujet âgé par des comorbidités contrairement à la population plus jeune qui présente des manifestations cliniques peu marquées.

Existe-t-il une sémiologie cutanée spécifique ou associée au COVID-19? Les manifestations cutanées restent rares au cours du COVID-19. Peu de cas ont été rapportés de manières sporadiques. Les premières données ont été recueillies par Recalcati et all. Sur 88 patients testés positifs, 20,4% avaient développé des manifestations cutanées [2]. Pour mémoire, on peut parler de dermatoses virales et de dermatoses paravirales réactionnelles. Actuellement, aucune publication n´a démontré l´effet cytopatogène et la présence du virus au niveau des lésions cutanées. C´est rappeler que certaines entités bien définies nosologiquement sont associées aux infections virales comme l´érythème polymorphe, l´urticaire, la vascularite, le syndrome de sweet. A la lecture de certaines publications [3,4], on s´aperçoit que les manifestations cutanées au cours du COVID-19 sont dominées par des éruptions érythématopapuleuses décrites comme un exanthème, une éruption PRG like ou une éruption varicelle like. Caractérisée par son apparition concomitante ou tardive après les signes respiratoires, l´éruption varicelle like se différencie de la varicelle par l´absence du prurit, des cicatrices et d´atteinte muqueuse. Pour l´urticaire souvent inaugurale, elle évolue vers une lente amélioration sous traitement symptomatique [4,5]. Pour l´érythème polymorphe (EP), l´origine médicamenteuse a été souvent discutée devant l´apparition tardive des lésions en post infectieux et après négativation de la PCR [6].

Enfin, il faut souligner l’absence de corrélation avec la sévérité de la maladie ainsi que l´incompréhension de la pathogenèse de la majorité des signes cutanés. En revanche le mécanisme des manifestations acrales et vasculaires a été attribué à une série de phénomènes thrombotiques tels que la coagulation intravasculaire disséminée (CIVD), la formation d´un thrombus hyalin, une dysrégulation immunologique ou une vascularite. En terme clinique, les manifestations sont variées et polymorphes: livédo, purpura, nécrose, avec une prédominance des pseudo-engelures ou pseudochilblain. D´apparition tardive chez des sujets jeunes souvent asymptomatiques avec des PCR négatives, les pseudo-engelures présentent des similitudes cliniques avec les lésions décrites dans les interféronopathies de type 1 de l´enfant. Certains auteurs les considèrent comme une réaction retardée de l´infection par le SARS-COV-2. Il s´agit probablement d´une réponse intérferon antivirale adaptée et signe d´une bonne évolution chez le sujet jeune. Elles sont exceptionnellement décrites chez des sujets âgés. Par ailleurs une revue récente suggère le rôle et la contribution de la voie STING (Stimulateur des gènes de l´interféron) dans les manifestations cutanées Kawasaki like chez les enfants COVID positifs [7].

Notre expérience au sein de l´hôpital Cheikh Khalifa où 144 patients ont été pris en charge, les signes cutanés observés chez deux patients admis en réanimation étaient une éruption érythématopapuleuse et papulovesiculeuse d´apparition tardive au niveau du tronc et des membres inférieurs. Les toxidermies au cours du COVID-19 sont secondaires aux traitements complémentaires (antibiotiques) et également aux traitements antiviraux et anti-inflammatoires toujours à l´essai. Une étude publiée par Zheng a rapporté des cas d´urticaire, vascularite et de prurit induits. Un cas de pustulose éxanthematique aiguë généralisée a été rapporté chez une patiente de 39 ans, 18 jours après le début du traitement par l´hydroxychloroquine. Bien qu´une amélioration ait été notée à l´arrêt du traitement, l´hypothèse que les éruptions soient en rapport avec l´infection virale a été soulevée [8,9]. Une autre difficulté s´est présentée aux dermatologues lors de cette pandémie concernant la prise en charge des patients testés positifs et qui sont sous immunosuppresseurs (Méthotréxate, Azathioprine, Cyclosporine, Biothérapie) pour des dermatoses inflammatoires chroniques tels que le psoriasis et la dermatite atopique. Ainsi un groupe d´expert a préconisé quelques recommandations en fonction du traitement utilisé, le risque engendré et la pathologie du patient [10].

## Conclusion

Au total, il faut signaler selon les différentes études la scission des signes cutanés en phase aiguë concomitants à la maladie (exanthème, urticaire, EP) et des pseudo-engelures et les signes vasculaires survenant à une phase plus tardive avec une signification et un mécanisme différents. Malgré le foisonnement des publications, le lien de causalité entre le SARS-COV-2 et les manifestations cutanées reste à démontrer par des études adaptées. En conséquence, les médecins et les dermatologues doivent être vigilants et conscients de ces signes afin de les reconnaitre et aider au diagnostic rapide de cette infection émergente.
